# Supplementary material for: The Impact of Temperature on Host–Parasite Interactions and Metabolomic Profiles in the Marine Diatom Coscinodiscus granii
Source: Plants (Basel). 2024 Dec 5;13(23):3415. doi: 10.3390/plants13233415 (PMC11644330; doi:10.3390/plants13233415)
Supplement: Supplementary file 1 [file plants-13-03415-s001.zip › plants-3289344-supplementary-1.pdf]

plants

The Impact of Temperature on Host–Parasite Interactions and Metabolomic Profiles in the Marine Diatom *Coscinodiscus granii*

Ruchicka Annie O'Niel, Georg Pohnert and Marine Vallet

Table S1. Study of infection rate monitored using starting cell densities of 200 cells per mL after seven days of incubation. The average infection rate were determined for biological replicates ($N = 3$).

Sample	Biological replicate	Total cell count	Number of infected cells (sporangia)	Number of healthy cells	Infection rate (infectivity) (%)
13^{0}C Algae – no parasite	1CG01	291	0	0	0
	1CG03	348	0	0	0
	1CG04	322	0	0	0
13°C Alga + parasite	1CG05	226	37	189	16.37168142
	1CG06	248	55	193	22.17741935
	1CG07	246	62	184	25.20325203
25^{0}C Algae – no parasite	1CG17	330	0	0	0
	1CG18	390	0	0	0
	1CG19	371	0	0	0
25^{0}C Alga + parasite	1CG22	380	46	334	12.10526316
	1CG23	367	28	339	7.629427793
	1CG24	334	17	317	5.089820359

Table S2. Selected differentially expressed metabolites in C. granii cells, treated with the marine parasite and grown at two temperatures. These compounds were elucidated by HR-MS, MS2, and library comparison using SIRIUS/GNPS/Compound Discoverer. Metabolites with proven identity by comparison with analytical standards are marked **in bold *.**

RT (min)	Chemical formula	Observed *m/z*	Mass deviation (ppm)	Adduct	Column	Compound name (SIRIUS and/or GNPS)	Database GNPS match link	Diagnostic fragments
4.42	$C_3H_7NO_3$	106.0505	6.79	$[M+H]^+$	Zic-hilic	**Serine *** CHEBI:17822	CCMSLIB00012476633	88.0758, 70.0655
4.17	$C_5H_9NO_3$	132.0663	6.49	$[M+H]^+$	Zic-hilic	L-4-hydroxyproline CHEBI:16231	CCMSLIB00006682247	86.0608, 68.0504
4.62	$C_5H_{10}O_2S$	135.0481	5.66	$[M+H]^+$	Zic-hilic	**DMSP *** CHEBI:16457	CCMSLIB00006716179	73.0293, 63.0272
4.33	$C_5H_{10}N_2O_3$	145.0609	-6.21	$[M-H]^-$	Zic-hilic	Glutamine CHEBI:28300	CCMSLIB00006121585	127.0505, 109.0401, 84.0448, 74.0239
4.09	$C_3H_9NO_4S$	156.0332	4.93	$[M+H]^+$	Zic-hilic	**Cysteinolic acid ***	CCMSLIB00008851455	138.0229, 74.0609, 56.0505, 58.0661, 60.0454
4.65	$C_7H_{15}NO_3$	162.1131	4.47	$[M+H]^+$	Zic-hilic	**Carnitine *** CHEBI:39547	CCMSLIB00006679452	103.0397, 85.0291, 60.0817
5.69	$C_6H_{14}N_4O_2$	173.1032	-6.54	$[M-H]^-$	Zic-hilic	Arginine CHEBI:32697	CCMSLIB00010102660	154.9465, 131.0817, 114.0553
5.97	$C_9H_{20}N_2O_2$	189.1607	5.23	$[M+H]^+$	Zic-hilic	Trimethyllysine CHEBI:17311	CCMSLIB00010103159	130.0872, 84.0815, 60.0818
4.28	$C_9H_{17}NO_4$	204.1239	4.46	$[M+H]^+$	Zic-hilic	**Acetylcarnitine *** CHEBI:73024	CCMSLIB00006678577	145.0504, 85.0291, 60.0817
4.13	$C_{10}H_{19}NO_4$	218.1395	4.134	$[M+H]^+$	Zic-hilic	Propanoyl-carnitine CHEBI:53210	CCMSLIB00006678782	159.0661, 85.0291, 60.0818
3.76	$C_{12}H_{10}N_4O_2$	243.0887	4.5	$[M+H]^+$	C18	**Lumichrome *** CHEBI:17781	CCMSLIB00010105809	216.0766, 200.0817, 172.0867
8.25	$C_{20}H_{30}O_2$	303.2327	-0.27	$[M+H]^+$	C18	**Eicosapentaenoic acid *** CHEBI:28364	CCMSLIB00012476635	161.1325, 135.1167, 121.1011, 109.1011, 81.0700, 67.0546
0.79	$C_{10}H_{13}N_5O_4$	268.1048	2.99	$[M+H]^+$	C18	Adenosine **CHEBI:16335**	CCMSLIB00005464314	136.0627, 115.0396, 85.0290, 57.034

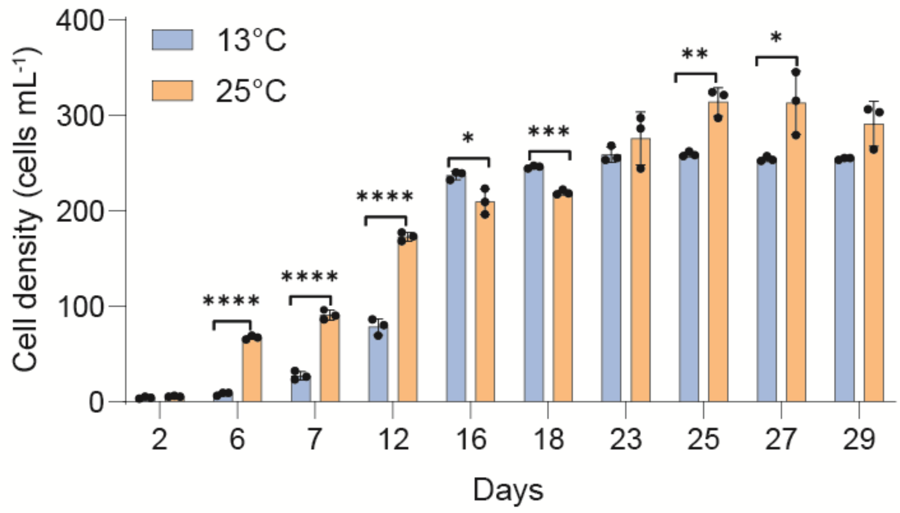

Figure S1. Cell density of diatom *Coscinodiscus granii* cultivated at 13 and 25°C. N = 3 and error bars indicate standard deviation.

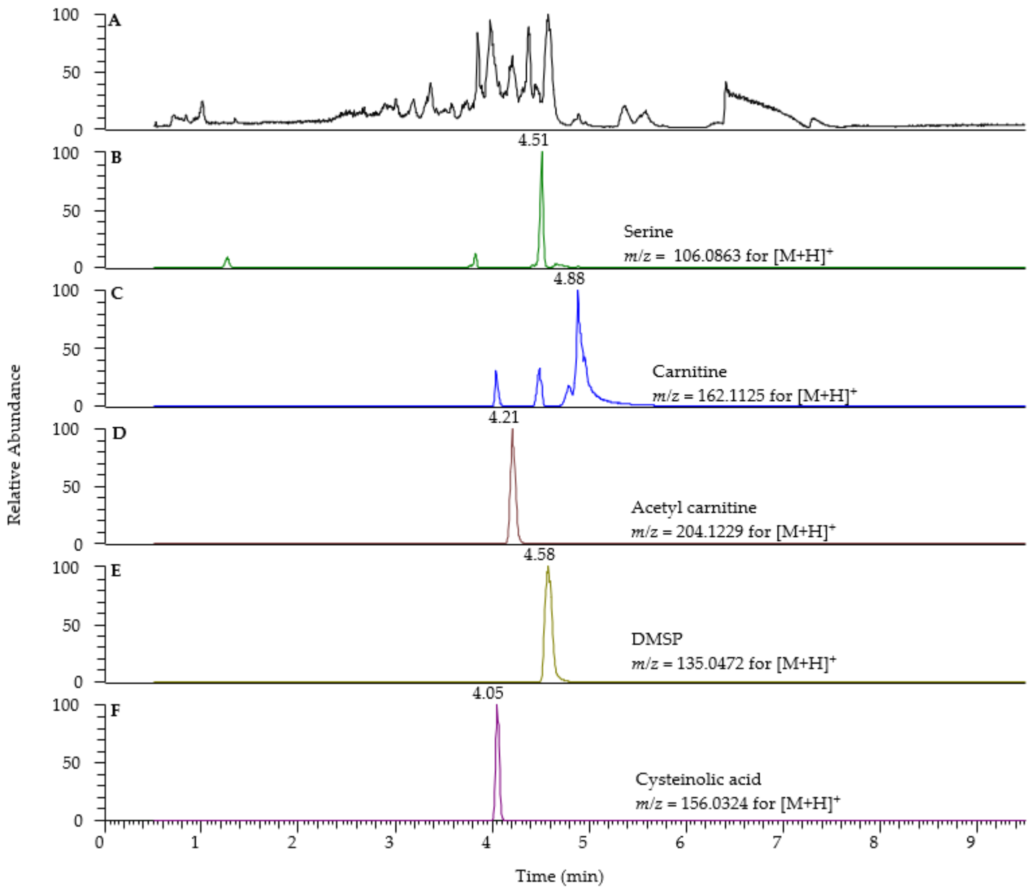

Figure S2. Chromatographic profile of zwitterionic metabolites from *C. granii* using UHPLC (Zic-Hilic column) with detection by ESI-HRMS. **A** Total Ion Chromatogram (TIC) in positive polarity of cell extract profile from QC sample, **B** Extracted Ion Chromatogram of Serine ion trace, *m/z* 106.0863, **C** Carnitine ion trace *m/z* 162.1125, **D** Extracted Ion Chromatogram of Acetyl carnitine, ion trace *m/z* 204.1229, **E** Extracted Ion Chromatogram of DMSP, ion trace *m/z* 135.0472, **F** Extracted Ion Chromatogram of Cysteinolic acid, ion trace *m/z* 156.0324.

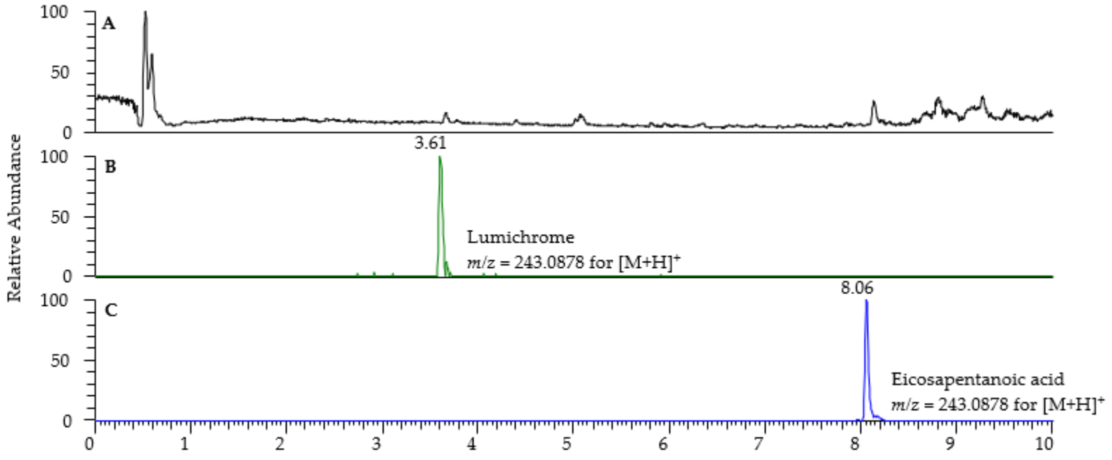

Figure S3. Chromatographic profile of non-polar metabolites from *C. granii* using UHPLC (C18 column) with detection by ESI-HRMS. **A** Total Ion Chromatogram (TIC) in positive polarity of cell extract profile from QC sample, **B** Extracted Ion Chromatogram of lumichrome, ion trace *m*/*z* 243.0878, **C** Extracted Ion Chromatogram of eicosapentaenoic acid, ion trace *m*/*z* 243.0878.

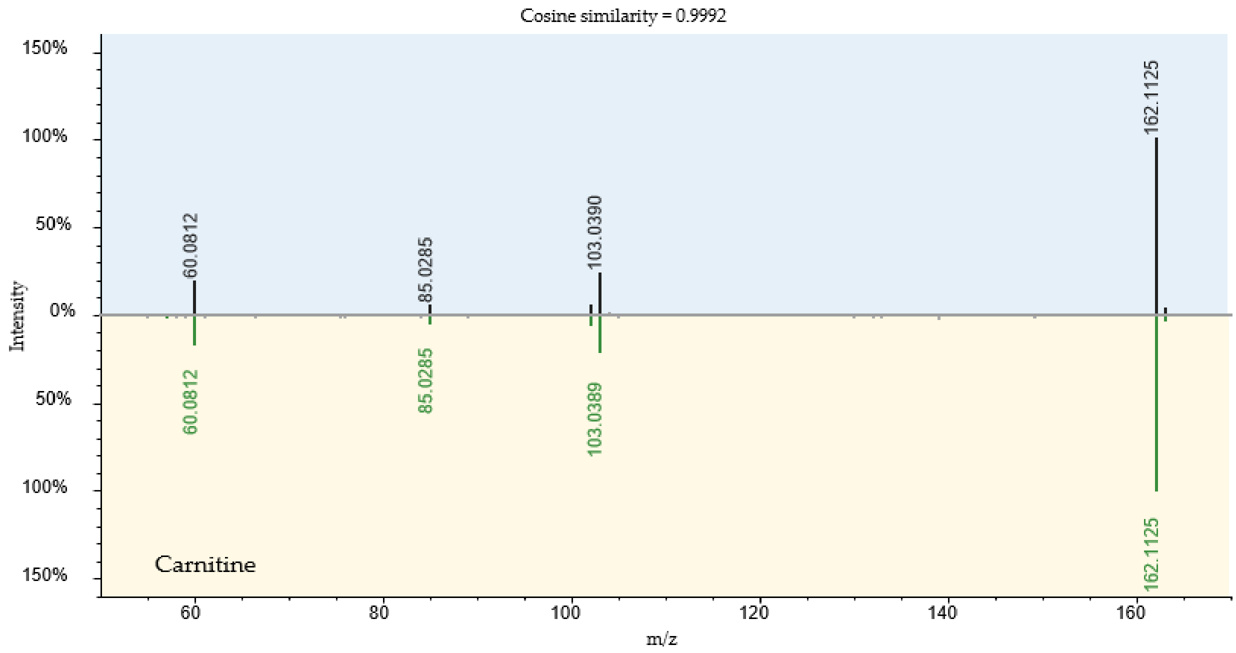

Figure S4. Comparison of MS/MS spectra of carnitine from coinjection studies of algal QC sample (upper plot) and reference standard (bottom plot). The link can be found here: CCMSLIB00012476631.

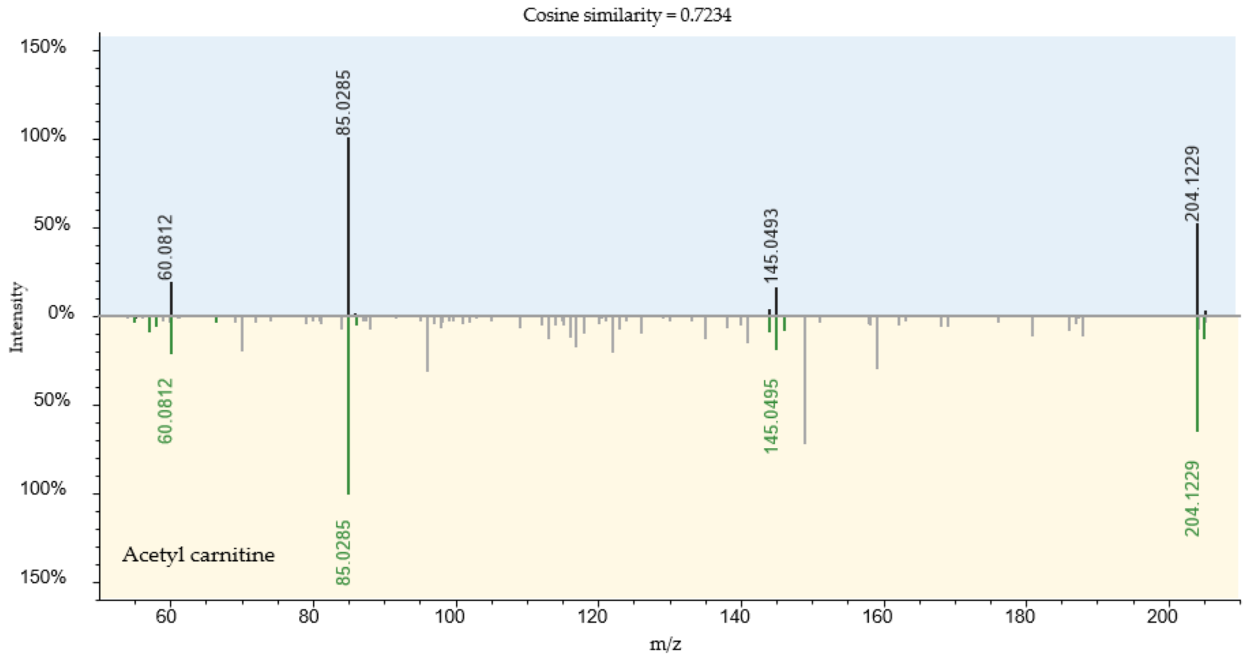

Figure S5. Comparison of MS/MS spectra of acetylcarnitine from coinjection studies of algal QC sample (upper plot) and reference standard (bottom plot). The link can be found here: CCMSLIB00012476630

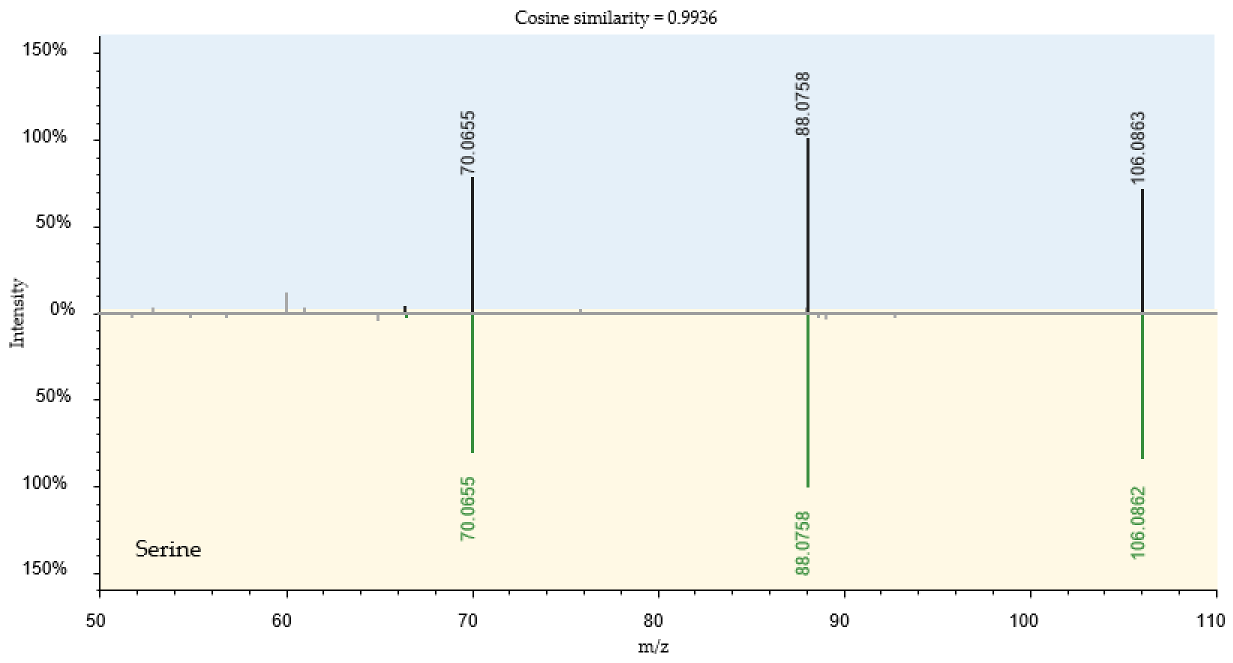

Figure S6. Comparison of MS/MS spectra of serine from coinjection studies of algal QC sample (upper plot) and reference standard (bottom plot). The link can be found here: CCMSLIB00012476633

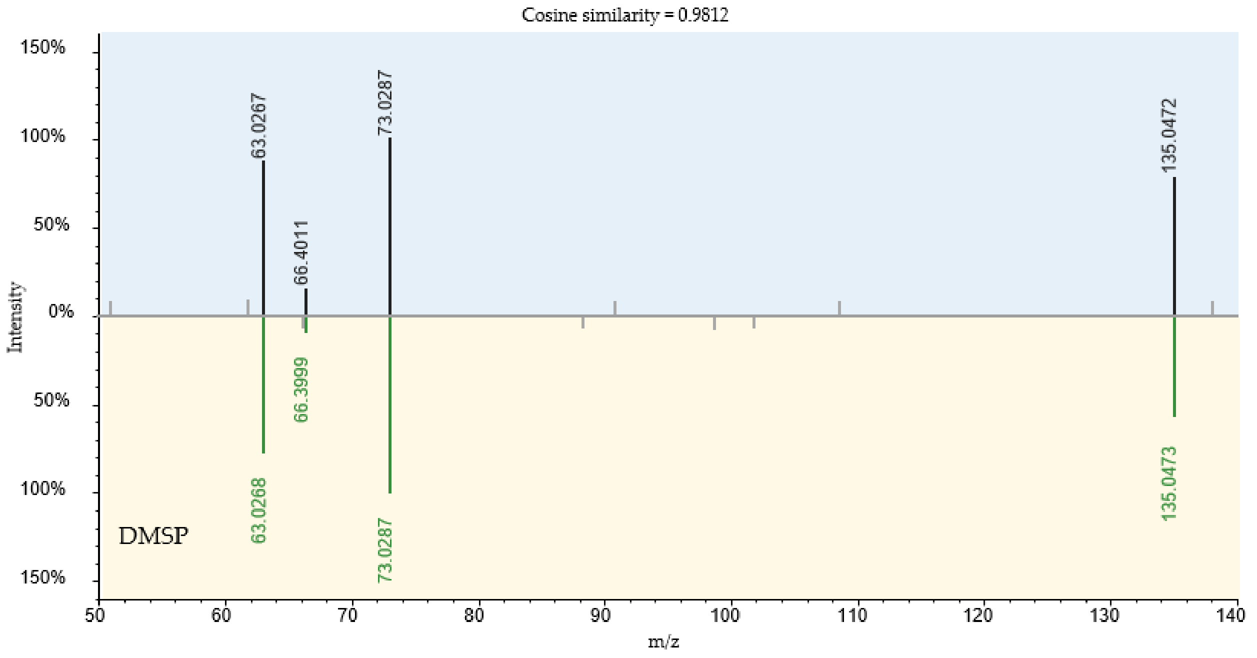

Figure S7. Comparison of MS/MS spectra of DMSP from coinjection studies of algal QC sample (upper plot) and reference standard (bottom plot). The link can be found here: CCMSLIB00012476634

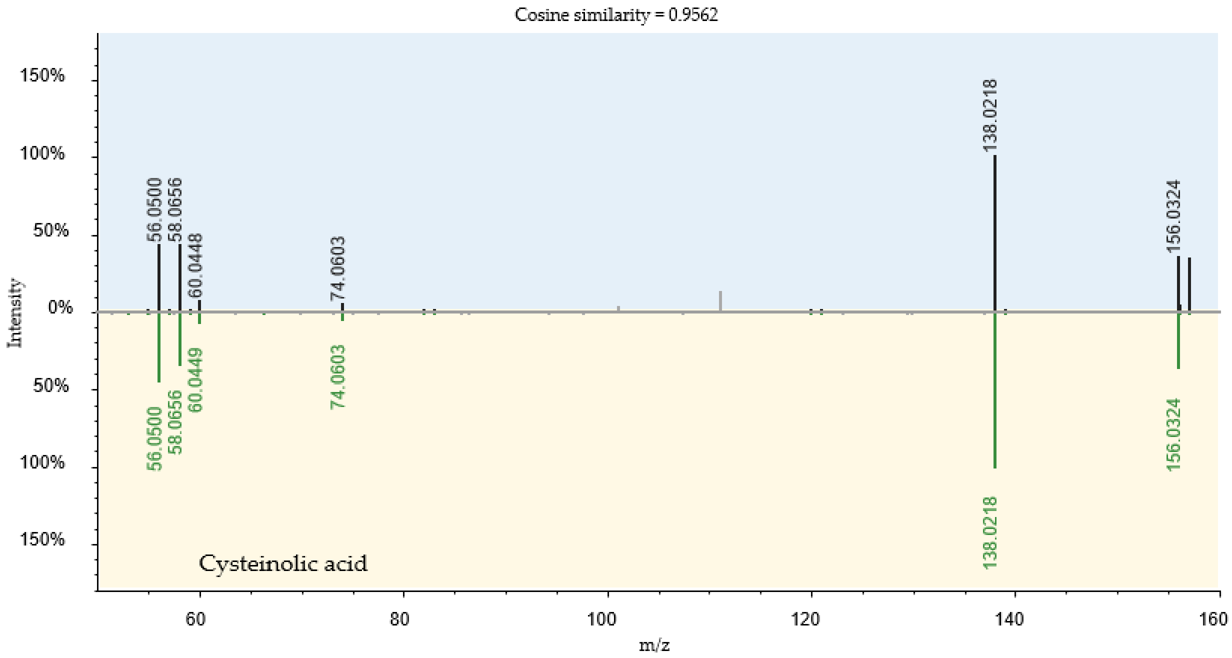

Figure S8. Comparison of MS/MS spectra of cysteinolic acid from coinjection studies of algal QC sample (upper plot) and reference standard (bottom plot). The link can be found here: CCMSLIB00012476632

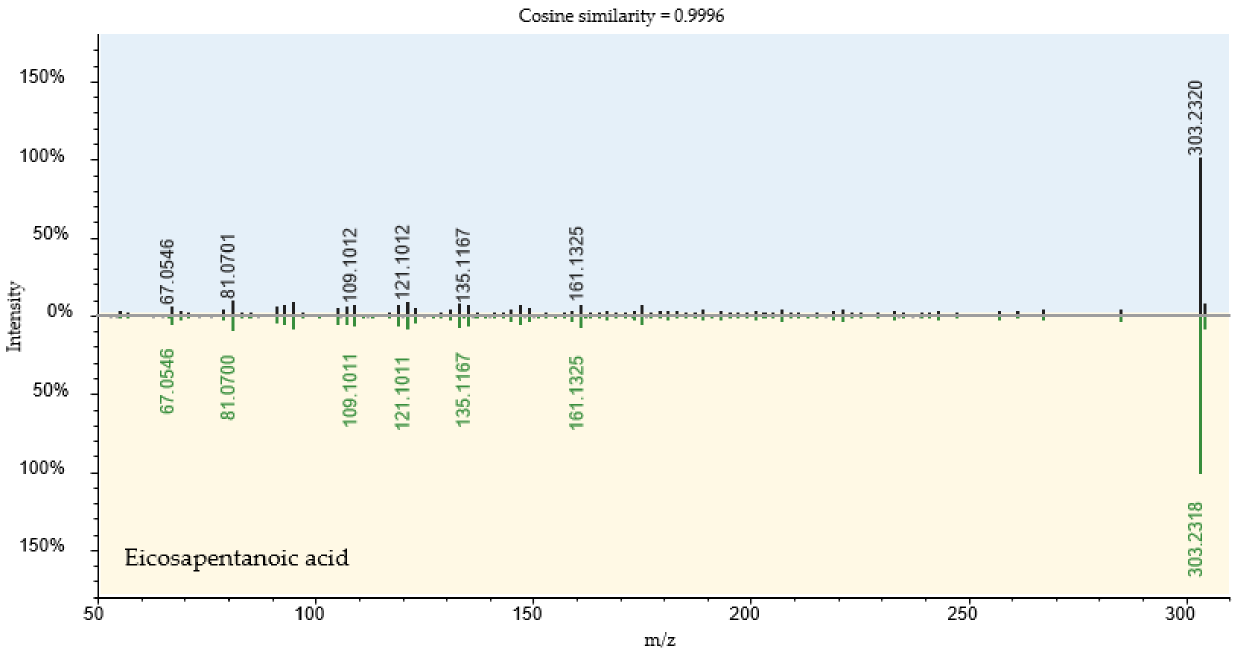

Figure S9. Comparison of MS/MS spectra of eicosapentaenoic acid from coinjection studies of algal QC sample (upper plot) and reference standard (bottom plot). The link can be found here: CCMSLIB00012476635

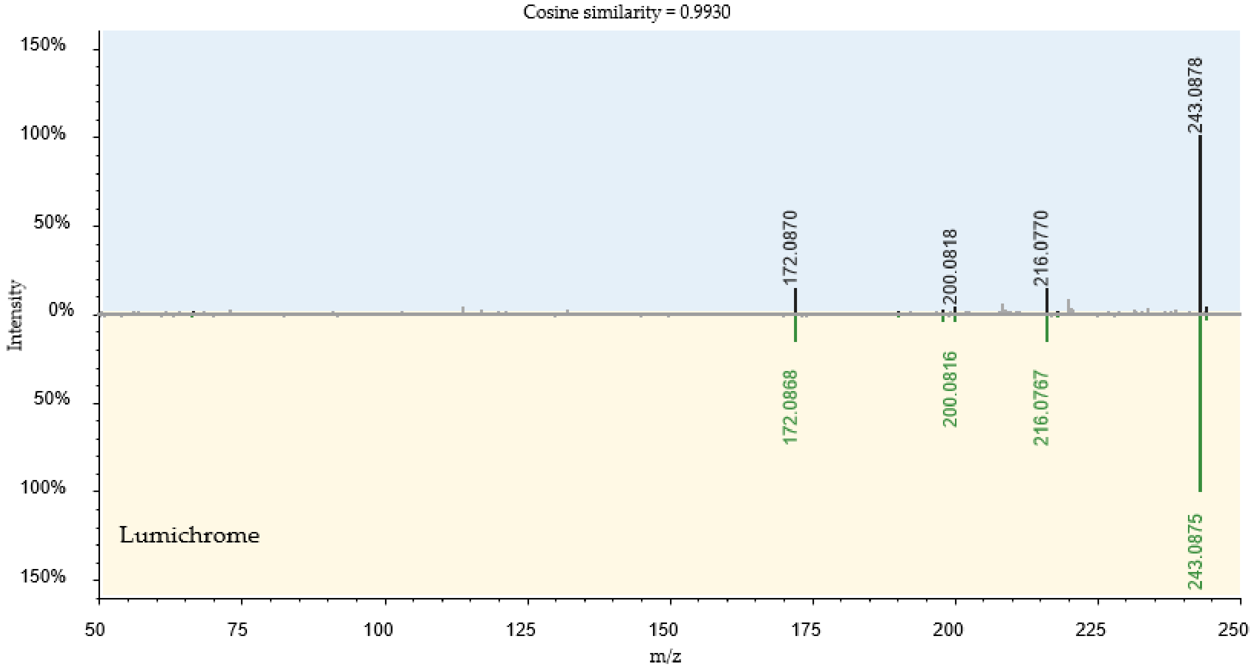

Figure S10. Comparison of MS/MS spectra of lumichrome from *C. granii* (upper plot) and reference standard (bottom plot) The link can be found here: CCMSLIB00012475013

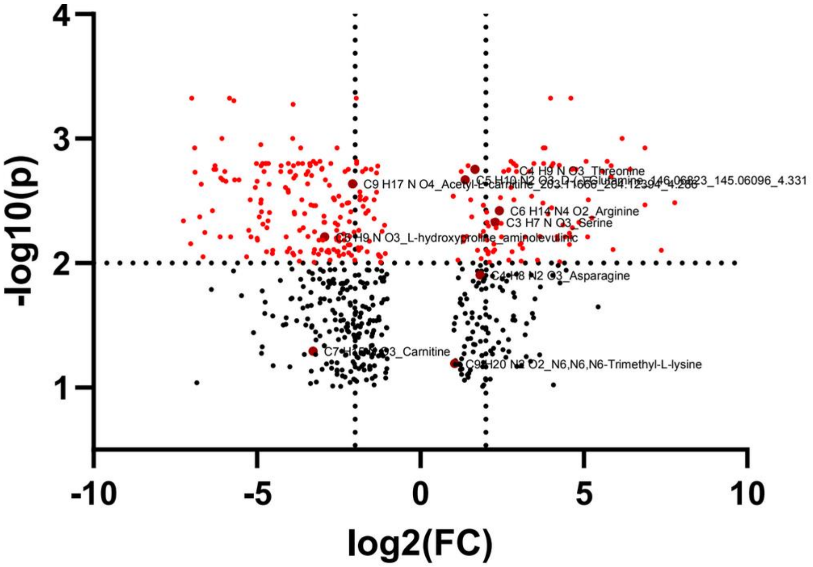

Figure S11. Volcano plot analysis of comparative metabolomics data for *C. granii* was extracted after the parasite treatment, cultured at 13°C and 25°C, and analyzed via LC-MS using ZIC-HILIC column. Red dots show statistically significant up and down-regulated metabolites in parasite-treated cultures grown at 13°C vs. 25°C for significant features with p-value < 0.05 and fold change > 2.

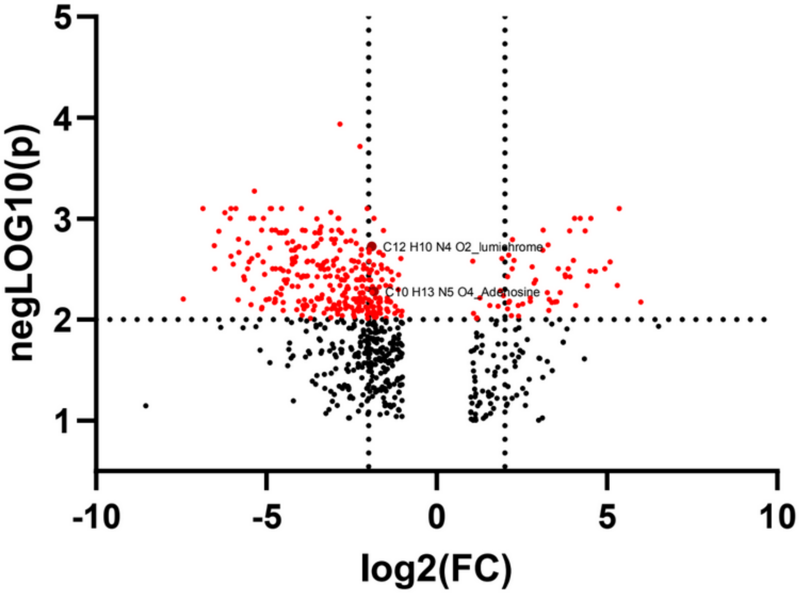

Figure S12. Volcano plot analysis of comparative metabolomics data for *C. granii* cells extracted after parasite treatment grown at 13°C and 25°C. The data was then analyzed via LC-MS using the C18 column. Red dots show statistically significant up and down-regulated metabolites in parasite-treated cultures grown at 13°C vs. 25°C for significant features with p-value < 0.05 and fold change > 2.

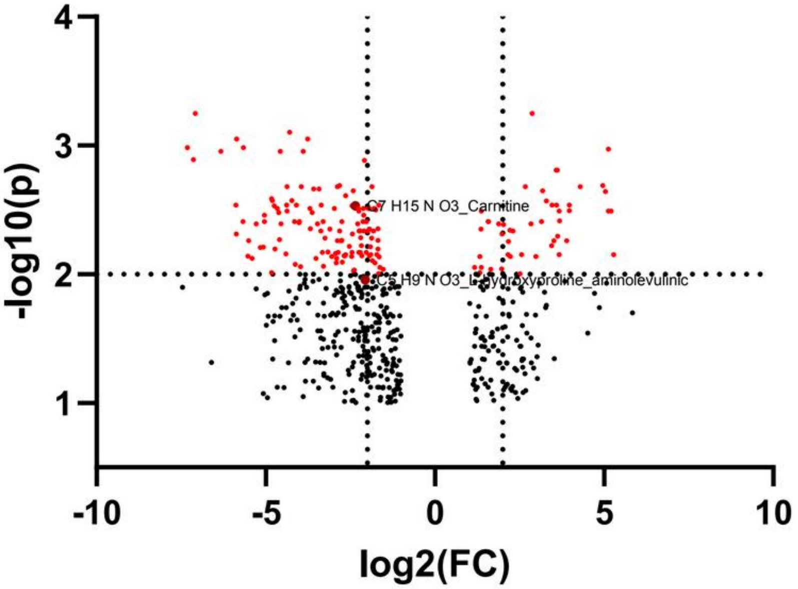

Figure S13. Volcano plot analysis of comparative metabolomics data for *C. granii* cells extracted after being cultured at 13°C and 25°C and analyzed via LC-MS using the ZIC-HILIC column. Red dots show statistically significant up and down-regulated metabolites in untreated cultures grown at 13°C vs. 25°C for significant features with p-value < 0.05 and fold change > 2.

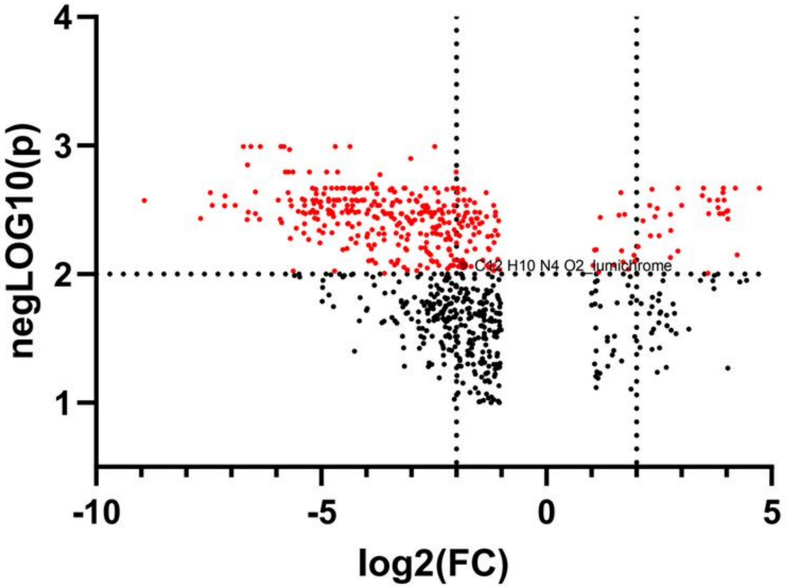

Figure S14. Volcano plot analysis of comparative metabolomics data for *C. granii* cells extracted after being cultured at 13°C and 25°C was analyzed via LC-MS using the C18 column. Red dots show statistically significant up and down-regulated metabolites in untreated cultures grown at 13°C vs. 25°C for significant features with p-value < 0.05 and fold change > 2.

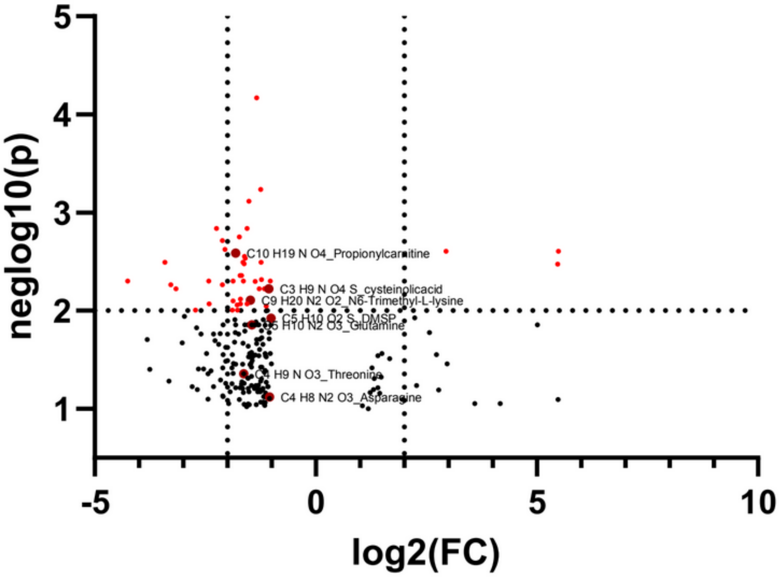

Figure S15. Volcano plot analysis of comparative metabolomics data for *C. granii* cells extracted after being cultured at 13°C and 25°C was analyzed via LC-MS using the ZIC-HILIC column. Red dots show statistically significant up- and down-regulated metabolites in untreated vs. parasite-treated cultures. Significant features with p-value < 0.05 and fold change > 2.

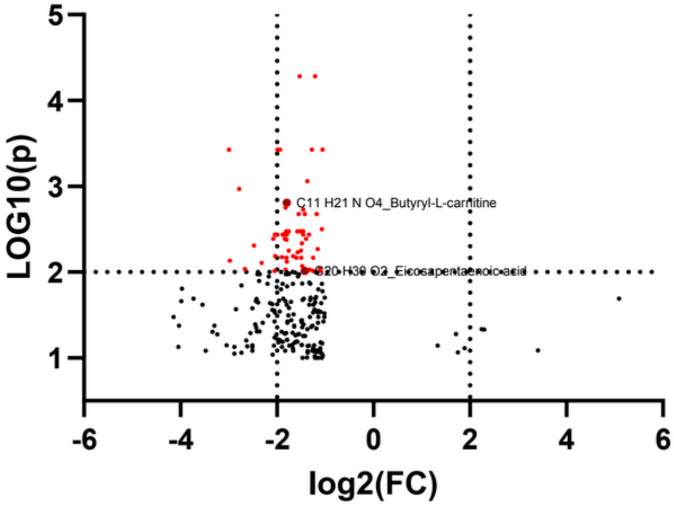

Figure S16. Volcano plot analysis of comparative metabolomics data for *C. granii* cells extracted after being cultured at 13°C and 25°C, and analyzed via LC-MS using the C18 column. Red dots show statistically significant up and down-regulated metabolites in untreated vs. parasite-treated cultures. Significant features with p-value < 0.05 and fold change > 2.

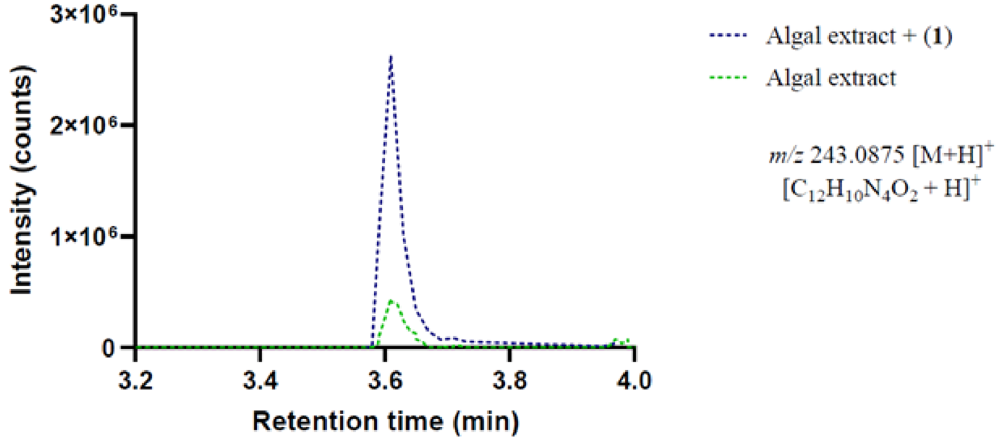

Figure S17. EIC (*m/z*) of *C. granii* (dashed green line; Algal extract) and the same extract spiked with the analytical standard lumichrome (dashed blue line; Algal extract + (**1**)).

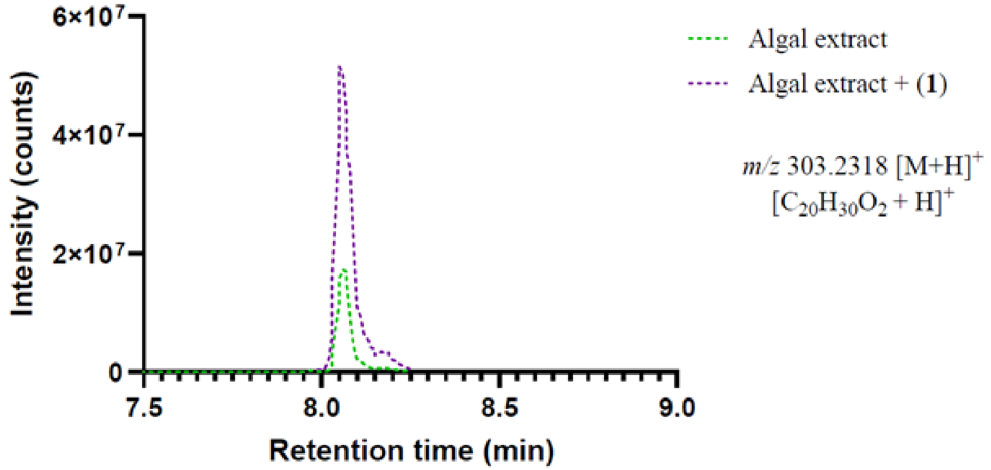

Figure S18. EIC (*m/z*) of *C. granii* (dashed green line; Algal extract) and the same extract spiked with the analytical standard eicosapentanoic acid (dashed purple line; Algal extract + (**1**)).

Table S3. Table summarizing ANOVA analysis to compare parasite treated diatom cultures infection rate at two temperatures 25°C and 13°C compared to control parasite untreated cultures.

**Two-way ANOVA**	**Ordinary**					
Alpha	0.05					
Source of Variation	% of total variation	P value	P value summary	Significant?		
Interaction	16.61	0.022	*	Yes		
parasite	19.18	0.016	*	Yes		
temperature	47.66	0.0014	**	Yes		
ANOVA table	SS	DF	MS	F (DFn	DFd)	P value
Interaction	5985	1	5985	F (1	8) = 8.026	P=0.0220
parasite	6912	1	6912	F (1	8) = 9.269	P=0.0160
temperature	17176	1	17176	F (1	8) = 23.03	P=0.0014
Residual	5966	8	745.8			
Difference between column means						
Mean of 13°C	286.3					
Mean of 25°C	362					
Difference between means	-75.67					
SE of difference	15.77					
95% CI of difference	-112.0 to -39.31					
Difference between row means						
Mean of - parasite	348.2					
Mean of + parasite	300.2					
Difference between means	48					
SE of difference	15.77					
95% CI of difference	11.64 to 84.36					
Interaction CI						
Mean diff	A1 - B1	-31				
Mean diff	A2 - B2	-120.3				
(A1 -B1) - (A2 - B2)	89.33					
95% CI of difference	16.62 to 162.0					
(B1 - A1) - (B2 - A2)	-89.33					
95% CI of difference	-162.0 to -16.62					
Rs of predicted Y vs. |residual|	0.3262					
P value (one tailed)	0.1563					
Passed (P > 0.05)?	Yes					
Normality of Residuals						
Test name	Statistics	P value	Passed normality test (alpha=0.05)?	P value summary		
D'Agostino-Pearson omnibus (K2)	1.716	0.4239	Yes	ns		
Anderson-Darling (A2*)	0.5793	0.1039	Yes	ns		
Shapiro-Wilk (W)	0.8892	0.115	Yes	ns		
Kolmogorov-Smirnov (distance)	0.2683	0.017	No	*		
Data summary						
Number of columns (temperature)	2					
Number of rows (parasite)	2					

Number of values	12
